# Association analysis of frost tolerance in rye using candidate genes and phenotypic data from controlled, semi-controlled, and field phenotyping platforms

**DOI:** 10.1186/1471-2229-11-146

**Published:** 2011-10-27

**Authors:** Yongle Li, Andreas Böck, Grit Haseneyer, Viktor Korzun, Peer Wilde, Chris-Carolin Schön, Donna P Ankerst, Eva Bauer

**Affiliations:** 1 Plant Breeding, Technische Universität München, Freising, Germany; 2 Biostatistics Unit, Technische Universität München, Freising, Germany; 3 KWS LOCHOW GMBH, Bergen, Germany; 4 Department of Mathematics, Technische Universität München, Garching, Germany

## Abstract

**Background:**

Frost is an important abiotic stress that limits cereal production in the temperate zone. As the most frost tolerant small grain cereal, rye (*Secale cereale *L.) is an ideal cereal model for investigating the genetic basis of frost tolerance (FT), a complex trait with polygenic inheritance. Using 201 genotypes from five Eastern and Middle European winter rye populations, this study reports a multi-platform candidate gene-based association analysis in rye using 161 single nucleotide polymorphisms (SNPs) and nine insertion-deletion (Indel) polymorphisms previously identified from twelve candidate genes with a putative role in the frost responsive network.

**Results:**

Phenotypic data analyses of FT in three different phenotyping platforms, controlled, semi-controlled and field, revealed significant genetic variations in the plant material under study. Statistically significant (*P *< 0.05) associations between FT and SNPs/haplotypes of candidate genes were identified. Two SNPs in *ScCbf15 *and one in *ScCbf12*, all leading to amino acid exchanges, were significantly associated with FT over all three phenotyping platforms. Distribution of SNP effect sizes expressed as percentage of the genetic variance explained by individual SNPs was highly skewed towards zero with a few SNPs obtaining large effects. Two-way epistasis was found between 14 pairs of candidate genes. Relatively low to medium empirical correlations of SNP-FT associations were observed across the three platforms underlining the need for multi-level experimentation for dissecting complex associations between genotypes and FT in rye.

**Conclusions:**

Candidate gene based-association studies are a powerful tool for investigating the genetic basis of FT in rye. Results of this study support the findings of bi-parental linkage mapping and expression studies that the *Cbf *gene family plays an essential role in FT.

## Background

Frost stress, one of the important abiotic stresses, not only limits the geographic distribution of crop production but also adversely affects crop development and yield through cold-induced desiccation, cellular damage and inhibition of metabolic reactions [1,2]. Thus, crop varieties with improved tolerance to frost are of enormous value for countries with severe winters. Frost tolerance (FT) is one of the most critical traits that determine winter survival of winter cereals [3]. Among small grain cereals rye (*Secale cereale *L.) is the most frost tolerant species and thus can be used as a cereal model for studying and improving FT [4,5]. After cold acclimation where plants are exposed to a period of low, but nonfreezing temperature, the most frost tolerant rye cultivar can survive under severe frost stress down to approximately -30°C [6]. Tests for evaluating FT can be generally separated into direct and indirect approaches. For direct approaches, where plants are exposed to both cold acclimation and freezing tests, plant survival rate, leaf damage, regeneration of the plant crown, electrolyte leakage, and chlorophyll fluorescence are often used as phenotypic endpoints [3]. For indirect approaches, where plants are exposed to only cold acclimation, the endpoints of water content [7], proline [8], and cold-induced proteins [9] are often used. The evaluation of FT can be conducted either naturally under field conditions or artificially in growth chambers, with both methods associated with advantages and disadvantages. Under field conditions, plant damage during winter is not only affected by low temperature stress *per se*, but also by the interaction of a range of factors such as snow coverage, water supply, and wind. Therefore, measured phenotypes are the result of the full range of factors affecting winter survival. Opportunities for assessing FT are highly dependent upon temperature and weather conditions during the experiment. In contrast, frost tests in growth chambers allow for a better control of environmental variation and are not limited to one trial per year. However, they are limited in capacity and may not correlate well with field performance. Therefore, it has been recommended to test FT under both natural and controlled conditions whenever possible [3].

FT is a complex trait with polygenic inheritance [6]. A large number of genes are up-and down-regulated when plants are exposed to cold/frost stress. Transcriptome analyses have estimated between 14% and 45% of the *Arabidopsis *genome to be cold responsive, dependent upon the cold treatment and other experimental factors [10-12]. Studies in wheat have shown between 5% and 8% of transcripts represented on microarrays to be regulated under cold stress [13,14]. More than 70 *Cold Responsive *(*COR*) genes in *Arabidopsis *are directly involved in cold/frost response with various functions such as enhancement of antioxidative mechanisms or stabilization of cellular membranes against dehydration damage [15,16]. The dehydrin gene family (*Dhn1-13*) is one group among the *COR *genes that has been characterized in barley [17]. Six members of the dehydrin gene family, including *HvDhn1 *and *HvDhn3*, were induced under mild frost stress in barley [18]. Expression of *COR *genes under cold stress in *Arabidopsis *is regulated through the binding of the *C-repeat binding factor *(*Cbf*) gene family to the *cis*-regulatory element DRE/CRT present in the promoter region of *COR *genes [2,19]. Most members of the *Cbf *gene family are closely linked and map to the *Fr2 *locus on homoeologous group 5, which coincides with a major quantitative trait locus (QTL) for FT in barley, diploid and hexaploid wheat, and meadow fescue [20-23]. Twelve members of the *Cbf *gene family have been assigned to the long arm of chromosome 5R in rye [24]. There is evidence that several members of the *Cbf *gene family are up-regulated by the transcription factor *Inducer of Cbf Expression 2 *(*Ice2*) under frost stress in hexaploid wheat and *Arabidopsis *[25,26]. Metabolite profiling in *Arabidopsis *has revealed between 311 (63%) and 434 (75%) metabolites altered in response to cold [27,28]. Among these, glucose, galactose, fructose, raffinose, sucrose, and xylose are involved in central carbohydrate metabolism and play a prominent role during reprogramming of metabolism under cold stress.

Identification of genes underlying traits of agronomic interest is pivotal for genome-based breeding. Due to methodological advances in molecular biology, plant breeders can now select varieties with favorable alleles via molecular markers, including single nucleotide polymorphisms (SNPs), identified in genes linked to desirable traits [29,30]. Whole-genome and candidate gene-based association studies have identified large numbers of genomic regions and individual genes related to a range of traits [31-34]. However, underlying population structure and/or familial relatedness (kinship) between genotypes under study have proven to be a big challenge, leading to false positive associations between molecular markers and traits in plants due to the heavily admixed nature of plant populations [35]. In response, several advanced statistical approaches have been developed for genotype-phenotype association studies, including genomic control [36], structured association [37], and linear mixed model-based methodologies [38,39]. The latter estimates population structure via a structure matrix and familial relatedness via a kinship matrix in a first step, and then includes these as covariates in a linear mixed model comprising the second step, thus arriving at phenotype-genotype association studies adjusting for population structure and kinship.

The main objective of this study was to identify SNP alleles and haplotypes conferring superior FT through candidate gene-based association studies performed in three phenotyping platforms, controlled, semi-controlled and field.

## Methods

### Plant material and DNA extraction

Plant material was derived from four Eastern and one Middle European cross-pollinated winter rye breeding populations: 44 plants from EKOAGRO (Poland), 68 plants from Petkus (Germany), 33 plants from PR 2733 (Belarus), 41 plants from ROM103 (Poland), and 15 plants from SMH2502 (Poland). To determine haplotype phase, gamete capture was performed by crossing between 15 and 68 plants of each source population to the same self-fertile inbred line, Lo152. Each resulting heterozygous S_0 _plant represented one gamete of the respective source population. S_0 _plants were selfed to obtain S_1 _families and these were subsequently selfed to produce S_1:2 _families, which were used in phenotyping experiments. For molecular analyses, genomic DNA of S_0 _plants was extracted from leaves according to a procedure described previously [40].

### Phenotypic data assessment and analyses

FT was measured in three phenotyping platforms: controlled, semi-controlled, and field. In the controlled platform, experiments were performed in climate chambers at -19°C and -21°C, in 2008 and 2009, respectively, at ARI Martonvásár (MAR), Hungary, using established protocols [41]. Briefly, seedlings were cold-acclimated in a six week hardening program with gradually decreasing temperatures from 15°C to -2°C. After that, plants were exposed to freezing temperatures within six days by decreasing the temperature from -2°C to -19°C or -21°C and then held at the lowest temperature for eight hours. After the freezing step, temperature was gradually increased to 17°C for regeneration. The ability of plants to re-grow was measured after two weeks using a recovery score, which ranged on a scale from 0: completely died, 1: little sign of life, 2: intensive damage, 3: moderate damage, 4: small damage, to 5: no damage. The light intensity was 260 μmol/m^2^s during the seedling growth and the hardening process, whereas the freezing cycle was carried out in dark. The experiment in 2008 contained 139 S_1 _families. The experiment in 2009 contained 201 S_1:2 _families, augmenting the same 139 S_1 _families from the experiment in 2008 with an additional 62 S_1:2 _families. Five plants of each S_1 _or S_1:2 _family were grown as one test unit with five replicates per temperature and year. Due to the limited capacity of climate chambers, genotypes were randomly assigned into three and four chambers in 2008 and 2009, respectively.

In the semi-controlled platform, experiments in the two years 2008 and 2009 were performed with 3 replicates per year at Oberer Lindenhof (OLI), Germany, using the same 139 S_1 _families and 201 S_1:2 _families. From each family a test unit of 25 plants was grown outdoors in wooden boxes one meter above the ground in a randomized complete block design (RCBD). In case of snowfall, plants were protected from snow coverage to avoid damage by snow molds. Two weeks after a frost period of 2-4 weeks with average daily temperatures around or below 0°C and usually frost at least during night with minimum temperatures as indicated in Additional File 1, % leaf damage was scored as the proportion of the 25 plants of each family that showed leaf damage (dry and yellow leaves). In order to keep the same sign as with the measurements in the controlled and field platforms, % leaf damage was replaced by % plants with undamaged leaves, calculated as 100% - % leaf damage. Outcomes were recorded in January, February, and April of 2008 for the 139 S_1 _families, and in February and March of 2009 for the 201 S_1:2 _families.

In the field platform, experiments were performed with the same 201 S_1:2 _families in five environments in 2009 (Kasan, Russia, KAS; Lipezk, Russia, LIP1; Minsk, Belarus, MIN; Saskatoon, Canada, two different fields, SAS1 and SAS2), and in one environment in 2010 (Lipezk, Russia, LIP2). Depending on the environment test units comprised 50-100 plants. The outcome, % survival, was calculated as the number of intact plants after winter divided by the total number of germinated plants before winter. RCBDs with 2 replicates were used for the SAS1 and SAS2 environments, while all other environments used the lattice design with 3 replicates. The climate data of the semi-controlled and field platforms are provided in Additional file 1.

To test phenotypic variation between genotypes, the same platform-specific models to be described for the SNP-FT association analyses were fitted for each platform omitting the SNP and population structure fixed effects. Within the controlled platform, separate models were fitted for each temperature and year combination, for the semi-controlled platform, separate models were fitted for each month of each year, and for the field platform separate models were fitted for each geographic location. The genetic variation was reported as the variance component corresponding to the random genotype effect in each model, with a *P*-value computed using the likelihood ratio test (LRT), a conservative estimate since the true asymptotic distribution of the LRT is a mixture of chi-square distributions [42].

### Population structure and kinship

In order to correct for confounding effects in the association studies, population structure and kinship was estimated based on 37 simple sequence repeat (SSR) markers that were chosen due to their experimental quality and map location as providing good coverage of the rye genome; details are found in [43]. Primers and PCR conditions were described in detail by Khlestkina et al. [44] for rye microsatellite site (RMS) markers and by Hackauf and Wehling [45] for *Secale cereale *microsatellite (SCM) markers. Fragments were separated on an ABI 3130xl Genetic Analyzer (Applied Biosystems Inc., Foster City, CA, USA) and allele sizes were assigned using the program GENEMAPPER (Applied Biosystems Inc., Foster City, CA, USA). Population structure was inferred from the 37 SSR markers using the STRUCTURE software v2.2, which is based on a Bayesian model-based clustering algorithm that incorporates admixture and allele correlation models to account for genetic material exchange in populations resulting in shared ancestry [46]. Briefly, the method assigned each individual to a predetermined number of groups (*k*) characterized by a set of allele frequencies at each locus, assuming that the loci are in Hardy-Weinberg equilibrium and linkage equilibrium. Ten runs for values of *k *ranging from two to eleven were performed using a burn-in period of 50,000 replications followed by 50,000 Markov Chain Monte Carlo iterations. Posterior probabilities of each *k *were averaged over the ten runs to determine the maximum posteriori *k*. The population structure matrix ***Q_STRUCTURE _***was estimated, providing for each of the 201 genotypes an estimate of the membership fraction in the *k *populations. The kinship matrix (***K***) was estimated from the same SSR markers using the allele-similarity method [47], which guarantees a positive semi-definite relationship matrix among the 201 genotypes, and was used for the covariance structure of the random genotype effects in the linear mixed model. For a given locus, the similarity index *S_xy _*between two genotypes was 1 when alleles were identical and 0 when alleles were different. *S_xy _*was averaged over the 37 loci and transformed and standardized as *Ŝ_xy _*= (*S_xy _*- *S_xymin_*)/(1 - *Ŝ_xymin_*), where *Ŝ_xymin _*is the minimum relationship in the matrix.

### SNP-FT association analyses

Twelve candidate genes *ScCbf2*, *ScCbf6*, *ScCbf9b*, *ScCbf11*, *ScCbf12*, *ScCbf14*, *ScCbf15*, *ScDhn1*, *ScDhn3*, *ScDreb2*, *ScIce2*, and *ScVrn1 *were selected for analysis due to their putative role in the FT network [17,20,24,25,48]. Details on candidate gene sequencing, SNP and insertion-deletion (Indel) detection, haplotype structure and linkage disequilibrium (LD) were described earlier [43] except for *ScDreb2*, which is described in Additional file 2. Indels were treated as single polymorphic sites, and for convenience polymorphic sites along the sequence in each gene were numbered starting with "SNP1" and are referred to in the text as SNPs instead of differentiating between SNPs and Indels.

SNP-FT associations in all platforms were performed using liner mixed models that evaluated the effects of SNPs with minor allele frequencies (MAF) > 5% individually, adjusting for population structure, kinship and platform-specific effects. A one stage approach was chosen for analysis which directly models the phenotypic raw data as the response. The general form of the linear mixed model for the three platforms was:

$$y=\beta_{1}+X_{\mathrm{SNP}}\beta_{SNP}+Q_{\mathrm{STRUCTURE}}\beta_{STRUCTURE}+X_{\mathrm{PLATFORM}}\beta_{PLATFORM}+Z_{\mathrm{PLATFORM}}\gamma_{PLATFORM}+Z_{\mathrm{GENOTYPE}}\gamma_{GENOTYPE}+\;\varepsilon ,$$

where *y *is the *n *× 1 vector of platform-specific phenotypes, ***X_SNP _***(*n *× *p*), ***Q_STRUCTURE _***(*n *× *q*) and ***X_PLATFORM _***(*n *× *k*) are design matrices for the fixed effects of SNPs, population membership and platform, respectively, and ***Z_PLATFORM _***(*n *× *m*) and ***Z_GENOTYPE _***(*n *× *l*) are the corresponding design matrices for the random effects of platform (described in detail below) and genotype, respectively, *β_1 _*is the intercept, *β_SNP _*is the allelic effect of the non-reference compared to reference allele (Lo152), and *β_STRUCTURE _*and *β_PLATFORM _*are the associated fixed effects for population structure and the platform-specific effects, respectively. If a platform contained random effects, these were accommodated by including a random effect *γ_PLATFORM _*~ N (0, ***D***σ^2^) with mean of 0, and variance-covariance matrix ***D***. The random genotype effect was similarly assumed to follow a Normal distribution, *γ_GENOTYPE _*~ N (0, ***K***σ^2^_g_), where ***K ***was the estimated kinship matrix and σ^2^_g _the variance component due to genotype. In order to account for kinship in the estimation of random genotype effects, γ*_GENOTYPE_*, the design matrix ***Z_GENOTYPE _***was multiplied by the cholesky-root of the kinship matrix. The residual error *ε *was assumed to comprise independent and identically distributed random Normal errors with mean of 0 and variance σ^2^, *ε *~ N (0, ***I***σ^2^).

Analyses of marker-FT associations were performed using the lme4 package [49] implemented in R [50]. Significance of individual SNP effects was assessed via the *t*-statistic performed at the two-sided alpha = 0.05 level. A multiple testing problem arises, which inflates the false positive rate of the study. A simple and common way to handle this problem is Bonferroni correction where the significance level is divided by the number of tests. However, the Bonferroni correction is too conservative and only suitable for independent tests, an assumption violated in this study due to a high LD between some of the SNPs as shown previously [43]. Therefore, the less stringent significance level of alpha = 0.05 is reported in the paper in order to retain candidates for further validation in upcoming experiments. The exact *P*-values are available in the Additional file 3 and can be adjusted for multiple testing. Empirical correlations between the 170 SNP-FT associations reported among the three phenotyping platforms were performed using Pearson's correlation based on the *t *values from the corresponding association tests. The genetic variation explained by an individual SNP or haplotype was calculated as 100 × ((σ^2^_g _- σ^2^_gSNP_)/σ^2^_g_), where σ^2^_g _is the genetic variation in the reduced model without an individual SNP and σ^2^_gSNP _is the model including an individual SNP [51]. This ad-hoc measure can result in negative estimates since variance components do not automatically decrease with more adjustment in a model as error sums of squares do; negative estimates were truncated to zero.

#### Controlled platform analyses

The outcome vector *y *was recovery score and the platform specific effect, *β_PLATFORM _*included the two years of measurement, 2008 and 2009, and two temperatures, -19 and -21°C. A common platform-specific random effect controlling for the seven chambers across the two years 2008 and 2009 was included in the model, *γ_PLATFORM _*~ N (0, ***I***σ^2^_chamber_), as it provided a more parsimonious model with the same goodness-of-fit as compared to a nested random effect structure within year. No additional explicit generation adjustment for S_1 _versus S_1:2 _families was included in the statistical model as these were confounded with the fixed effect adjustment for year and the random chamber effects, and hence could not be additionally estimated. In other words, the generation effect was assumed implicitly adjusted for by other year effects in the model.

#### Semi-controlled platform analyses

The outcome vector *y *was % plants with undamaged leaves measured repeatedly over three months (January, February, and April) in 2008 and two months (February, March) in 2009. Linear mixed models were formulated for individual test units, each comprising approximately 25 plants. The platform-specific fixed effect vector, *β_PLATFORM_*, included three terms: a year effect, an overall linear trend in time for the three months in 2008 and two months in 2009, and the interaction of year and linear trend in time. Three platform-specific random effects (vector *γ_PLATFORM_*) were used: replication, a random intercept and a random trend with month. The replication random effect was assumed uncorrelated with the random intercept and trend.

#### Field platform analyses

The outcome vector *y *was % survival and the platform-specific fixed effect *β_PLATFORM _*included indicator variables for the six environments, five environments in 2009 and one in 2010. Platform-specific random effects included a block effect nested within environments arising from the lattice design.

### Haplotype-FT association analyses and gene × gene interaction

Haplotype phase was determined by subtracting the common parent Lo152 alleles and haplotypes were defined within each candidate gene using DnaSP v5.10 [52]. Haplotype-FT associations were performed using candidate gene haplotypes with MAF > 5%. The same platform-specific statistical models controlling for population structure, kinship, and platform-specific effects were used to test associations between haplotypes of the respective candidate genes and FT. For these analyses *β_hap _*replaced *β_SNP _*as a measure of the haplotype effect of the non-reference compared to the reference haplotype Lo152. First, significant differences between haplotypes of one gene were assessed using the likelihood ratio test. If the overall statistic was significant, individual haplotype effects were tested against the reference haplotype Lo152 via *t*-tests. Based on haplotype information gene × gene interactions were assessed using the likelihood ratio test, comparing the full model with main effects plus interaction to the reduced model with main effects only.

## Results

### Phenotypic data analyses

**Figure 1:**
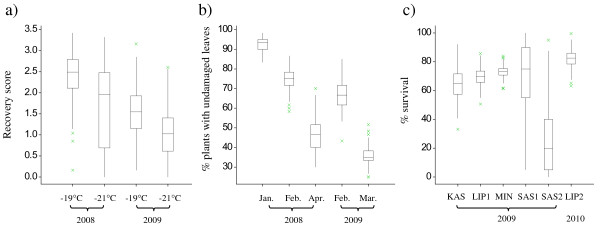
**Phenotypic variation in three phenotyping platforms: a) controlled platform, b) semi-controlled platform, and c) field platform**. The values are the average phenotypic raw value of replicates for each genotype. Boxes indicate the range of the middle 50% of the data with a horizontal line representing the median and vertical lines beyond the boxes indicate the upper and lower 25% of the data. Outliers are represented by crosses.

Phenotypic assessments of FT were carried out in 12 environments from three different phenotyping platforms. Phenotypic data were analyzed separately in each environment (Figure 1). Genotypic variation for FT was significant at both temperatures for both years in the controlled platform (*P *< 0.001). Recovery scores ranged from a median near 2.5 (between intensive and moderate damage) at -19°C in 2008 to a median near 1.0 (little sign of life) at -21°C in 2009. As expected, recovery scores were higher at -19°C than at -21°C in the same year but were lower in 2009 than in 2008 probably due to different generations of rye material (S_1 _vs S_1:2 _families). The high variability at -21°C in 2008 might have been induced by substantial variation between chambers. In the semi-controlled platform, genotypic variation for FT was significant during all months for both years (*P *< 0.01). Linear decreasing trends were observed during each year which was expected since those were longitudinal data and thus the damaged portions of plants increased during the progression of winter. In the field platform, genotypic variation for FT was significant (*P *< 0.05) in four (LIP1, LIP2, SAS1, and SAS2) of the six environments (*P *< 0.05). Compared to other environments, SAS1 and SAS2 showed a better differentiation for FT among genotypes, ranging from 5% to 100% with a median of 75% and 0% to 95% with a median of 20% survival rate, respectively. The large difference of survival rate between SAS1 and SAS2 was probably due to different altitudes and consequently different severity of frost stress.

### Population structure and kinship

**Figure 2:**
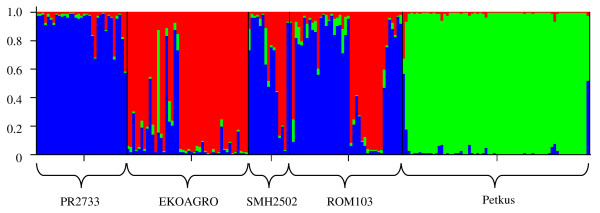
**Population structure based on genotyping data of 37 SSR markers**. Each genotype is represented by a thin vertical line, which is partitioned into *k *= 3 colored segments that represent the genotype's estimated membership fractions shown on the y-axis in *k *clusters. Genotypes were sorted according to populations along the x-axis and information on population origin is given.

Based on the STRUCTURE analysis, *k *= 3 was the most probable number of groups. Populations PR2733 (Belarus) and Petkus (Germany) formed two distinct groups while populations EKOAGRO, SMH2502, and ROM103 (all from Poland) were admixed in the third group with shared membership fractions with population PR2733 (Figure 2). This could likely be attributed to seed exchange between the populations from Belarus and Poland. The relatedness among the 201 genotypes estimated from the allele-similarity kinship matrix ranged from 0.11 to 1.00 with a mean of 0.37. Compared to the Eastern European populations, genotypes from Petkus showed a higher relatedness among each other with a mean of 0.53.

### Association analyses

**Figure 3:**
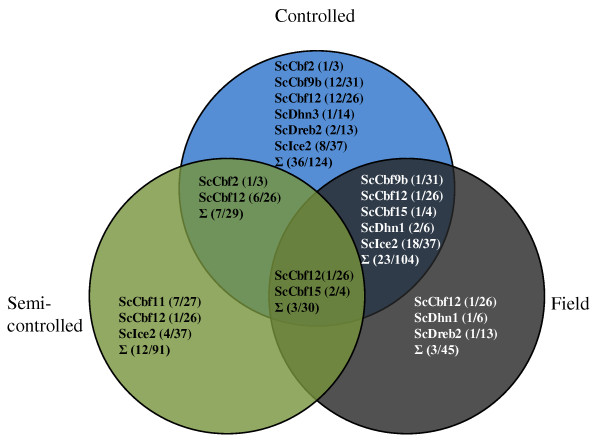
**Venn diagram of SNPs from candidate genes significantly (*P *< 0.05) associated with frost tolerance in three phenotyping platforms**. The first and second numbers in each bracket are the number of significant SNPs and total number of SNPs in each candidate gene.

SNP-FT associations were performed using 170 SNPs from twelve candidate genes. In the controlled platform, 69 statistically significant SNPs were identified among nine genes: *ScCbf2*, *ScCbf9b*, *ScCbf11*, *ScCbf12*, *ScCbf15*, *ScDhn1*, *ScDhn3*, *ScDreb2*, *ScIce2 *(all *P *< 0.05; Figure 3). In the semi-controlled platform, 22 statistically significant (*P *< 0.05) SNPs were identified among five genes: *ScCbf2*, *ScCbf11*, *ScCbf12*, *ScCbf15*, and *ScIce2*. In the field platform, 29 statistically significant (*P *< 0.05) SNPs were identified among six genes: *ScCbf9b*, *ScCbf12*, *ScCbf15*, *ScDhn1*, *ScDreb2*, and *ScIce2*. Eighty-four SNPs from nine genes were significantly associated with FT in at least one of the three platforms, and 33 SNPs from six genes were significantly associated with FT in at least two of the three platforms. Across all three phenotyping platforms, two SNPs in *ScCbf15 *and one SNP in *ScCbf12 *were significantly associated with FT; all of these three SNPs are non-synonymous, causing amino acid replacements. No SNP-FT associations were found for SNPs in *ScCbf6*, *ScCbf14*, and *ScVrn1*. Full information on SNP-FT associations for all platforms can be found in Additional file 3.

**Figure 4:**
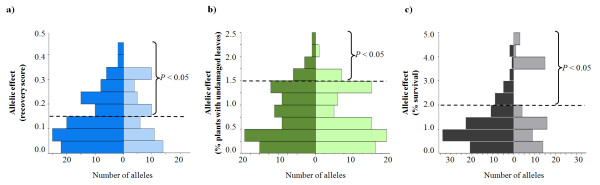
**Distribution of allelic effects (*β_SNP_*) of SNP - frost tolerance associations in a) controlled, b) semi-controlled, and c) field platforms**. The left and right hand side bars in a), b) and c) represent alleles with negative and positive effects relative to the Lo152 reference allele, respectively. The significance threshold (*P *< 0.05) for each platform is indicated by a dashed line.

**Figure 5:**
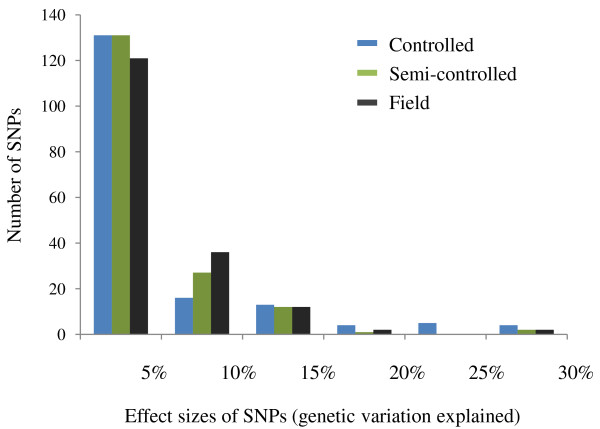
**Distributions of effect sizes of SNPs in three phenotyping platforms**. Effect sizes are displayed as genetic variation explained by individual SNPs.

Allelic effects (*β_SNP_*) of the 170 SNPs studied were relatively low, ranging from -0.43 to 0.32 for recovery scores in the controlled platform, -2.17% to 2.44% for % plants with undamaged leaves in the semi-controlled platform, and -3.66% to 4.30% for % survival in the field platform (Figure 4). 45.5% of all significant SNPs found in at least one platform had positive allelic effects, indicating the non-reference allele conveyed superior FT to the reference allele. The largest positive *β_SNP _*among the 170 SNPs in the field platform was observed for SNP7 in *ScIce2 *(*β_SNP _*= 4.30). This favorable allele was present predominantly in the PR2733 population (55.2%), and occurred at much lower frequency in the other four populations (EKOAGRO: 4.7%, Petkus: 0%, ROM103: 7.1% and SMH2502: 6.7%). The proportion of genetic variation explained by individual SNPs ranged from 0% to 27.9% with a median of 0.4% in the controlled platform, from 0% to 25.6% with a median of 1.2% in the semi-controlled platform, and from 0% to 28.9% with a median of 2.0% in the field platform (Figure 5). These distributions were highly concentrated near zero.

Empirical correlations of the SNP-FT association results, in terms of *t *values, between the three phenotyping platforms were moderate to low. The highest correlation coefficient was observed between the controlled and semi-controlled platform with *r *= 0.56, followed by correlations between the controlled and field platform with *r *= 0.54, and the semi-controlled and field platform with *r *= 0.18. When correlations were restricted to the significant SNPs, slightly higher correlation coefficients were observed with *r *= 0.64 between the controlled and semi-controlled platform, *r *= 0.66 between the controlled and field platform, and *r *= 0.34 between the semi-controlled and field platform.

Haplotype-FT associations were performed using 30 haplotypes (MAF > 5%) in eleven candidate genes. Because only one haplotype in *ScDhn1 *had a MAF > 5%, *ScDhn1 *was excluded from further analysis. Large numbers of rare haplotypes (MAF < 5%) were found in *ScCbf9b *(N = 62) and *ScCbf12 *(N = 22) resulting in large numbers of missing genotypes (87.9% and 61.3%) for the association analysis. Haplotypes 2, 3, and 4 in *ScCbf2 *were significantly (*P *< 0.05) associated with FT in the controlled platform. For haplotypes 1 and 2 in *ScCbf15 *and haplotype 1 in *ScIce2*, significant associations (*P *< 0.05) were found across two and three platforms, respectively (Table 1). Haplotype effects (*β_Hap_*) were relatively low and comparable to the allelic effects (*β_SNP_*) ranging from -0.31 to 0.49 (recovery score), -1.71% to 2.74% (% plants with undamaged leaves), and -3.32% to 3.47% (% survival) in the controlled, semi-controlled and field platform, respectively. The highest positive effect on survival rate was observed for haplotype 1 of *ScIce2 *in the field platform, implicating this haplotype as the best candidate with superior FT. This favorable haplotype was present mainly in the PR2733 population (35.7%), occurring in much lower frequencies in the other four populations (0.0% in EKOAGRO, 0.0% in Petkus, 5.3% in ROM103, and 6.7% in SMH2503). The proportion of genetic variation explained by the haplotypes ranged from 0% to 25.7% with a median of 1.6% in the controlled platform, from 0% to 17.6% with a median of 1.4% in the semi-controlled platform, and from 0% to 9.3% with a median of 4.8% in the field platform.

**Table 1:** Summary of haplotypes significantly associated with frost tolerance in at least one platform, their haplotype effects, and percent genetic variation explained by the haplotypes

Candidate gene	**Name of haplotype**^**a**^	**Controlled (recovery score 0-5)**^**b**^			Semi-controlled (% plants with undamaged leaves)			Field (% survival)		
		
		***P*-value**^**c**^	***β***_***Hap***_	% genetic variation explained	*P*-value	*β_Hap_*	% genetic variation explained	*P*-value	*β_Hap_*	% genetic variation explained
*ScCbf2*	Overall**^d^**	**< 0.001**	-	25.7	0.21	-	16.3	0.40	-	5.0
	2	**0.04**	-0.11	-	0.51	-0.51	-	0.73	-0.51	-
	3	**< 0.001**	0.49	-	0.19	1.36	-	0.12	3.32	-
	4	**< 0.001**	-0.31	-	0.12	-1.43	-	0.74	0.57	-
*ScCbf15*	Overall	**< 0.01**	-	0.6	0.09	-	17.6	0.09	-	4.4
	1	**< 0.01**	-0.22	-	**0.04**	-1.69	-	0.06	-3.32	-
	2	**< 0.01**	-0.21	-	0.13	-0.92	-	**0.04**	-2.59	-
*ScIce2*	Overall	**0.04**	-	4.8	**0.02**	-	13.3	0.13	-	8.1
	1	**< 0.01**	0.29	-	**< 0.01**	2.74	-	**0.02**	3.47	-

^a ^Haplotypes with minor allele frequency (MAF) > 5%

^b ^0: completely died. 1: little sign of life. 2: intensive damage. 3: moderate damage. 4: small damage. 5: no damage

^c ^*P*-values < 0.05 are printed in bold

^d ^All haplotypes (MAF > 5%) within a candidate gene

**Figure 6:**
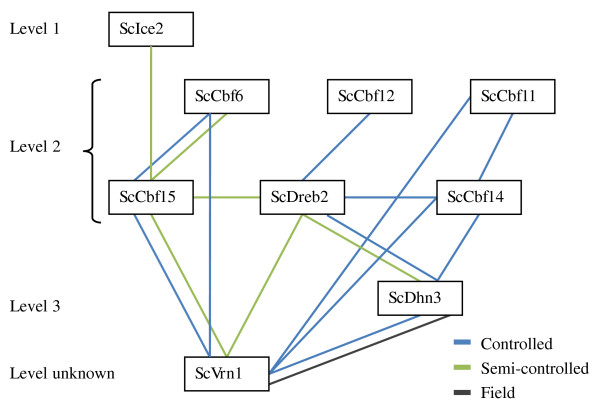
**Significant (P < 0.05) gene × gene interactions for frost tolerance in three phenotyping platforms**. Candidate genes are sorted in three levels according to the frost responsive cascade [19]. The level where *ScVrn1 *belongs to is still unknown.

Out of all possible gene × gene interactions tested on the basis of haplotypes, eleven, six, and one were significantly (*P *< 0.05) associated with FT in the controlled, semi-controlled and field platforms, respectively. *ScCbf15 *× *ScCbf6*, *ScCbf15 *× *ScVrn1*, *ScDhn3 *× *ScDreb2*, and *ScDhn3 *× *ScVrn1 *were significantly associated with FT across two platforms, none were significantly associated with FT across all three platforms (Figure 6).

## Discussion

FT is a complex trait with polygenic inheritance. While the genetic basis of FT has been widely studied in cereals by bi-parental linkage mapping and expression profiling, exploitation of the allelic and phenotypic variation of FT in rye by association studies has lagged behind [20,21,24]. This study reports the first candidate gene-based association studies in rye examining the genetic basis of FT.

Statistically significant SNP-FT associations were identified in nine candidate genes hypothesized to be involved in the frost responsive network among which the transcription factor *Ice2 *is one of the key factors. The function of *Ice2 *was characterized both in wheat and *Arabidopsis *[25,26]. Over-expression of *TaIce2 *and *AtIce2 *in transgenic *Arabidopsis *plants resulted in increased FT of transgenic plants and was associated with higher expression levels of the *Cbf *gene family. Using electrophoresis mobility shift assays, Badawi et al. [25] further showed that *TaIce2 *binds to the promoter region of *TaCbf9*. We were not able to detect interaction between *ScIce2 *and *ScCbf9b *probably due to the large number of rare haplotypes (MAF < 5%) in *ScCbf9b *resulting in missing genotypes (87.9%) and thus in insufficient statistical power to identify gene × gene interaction. However, in the rye homolog *ScIce2*, we detected 30 out of 37 SNPs with high LD (average *r^2 ^*= 0.85) which were significantly associated with FT. Our results support the findings of expression studies that *Ice2 *is one of the key elements in the frost responsive network. Given that these 30 SNPs are all located in the first intron of the gene, they are unlikely to be functional. However, it is possible that they are in LD with functional polymorphisms located in the coding sequence (CDS) of the gene which we have not investigated due to a lack of rye sequences in GenBank for primer design. The favorable allele of SNP7 in *ScIce2 *had a relatively large allelic effect on FT in the controlled and field platforms when compared to other SNPs in this study. This allele was present predominantly in the PR2733 population while entirely absent in the Petkus population. Thus, this SNP might facilitate marker-assisted backcrossing to introgress favorable genomic regions into the Petkus population, thereby improving FT of current breeding materials.

The *Cbf *gene family, regulated by *Ice2*, belongs to the family of *APETALA2 *(*AP2*) transcription factors, some of which are closely linked in cereals and map to the FT locus *Fr2 *on homoeologous group 5 of the *Triticeae *[20-22]. Expression studies have revealed that the *Cbf *gene family is involved in the frost responsive network in diverse species [10,24,53]. In this study, seven *Cbf *genes were investigated and statistically significant associations were found in at least one platform for *ScCbf2*, *ScCbf9b*, *ScCbf11*, *ScCbf12*, and *ScCbf15 *but not for *ScCbf6 *and *ScCbf14*. This confirms previous studies that not all members of the *Cbf *gene family are involved in the frost responsive network [24,53]. In *ScCbf2*, a 200 bp Indel was highly associated (*P *= 6.27e^-5^) with FT in the controlled platform and explained a high proportion of the genetic variation in the controlled (25.7%) and semi-controlled (16.3%) platforms. It is noteworthy that this 200 bp Indel in the promoter of *ScCbf2 *contained two MYB and one MYC *cis*-elements. In wheat the presence of MYB and MYC elements has been shown to affect the binding specificity of TaIce41 (wheat homolog of ScIce2) and consequently the expression level of the *TaCbf *gene family [25]. Expression studies are needed to investigate the effect of multiple binding sites for ScIce2 in *Cbf *gene promoters on the expression level of *Cbf *genes. A study in *Triticum monococcum *suggested that polymorphisms in *TmCbf12*, *TmCbf14*, and *TmCbf15 *are the most likely explanation for observed differences in FT [22]. Among the four significantly associated *Cbf *genes in our study, one SNP in *ScCbf12 *and two SNPs in high LD (*r^2 ^*= 0.73) in *ScCbf15 *were significantly associated with FT across all three platforms. Given that these three SNPs are all non-synonymous, leading to amino acid exchanges in the CDS of their respective genes, they are good candidates for functional genetic studies. In a recent study, Fricano et al. [54] found two SNPs located in the 3'-untranslated region of *HvCbf14 *significantly associated with FT in wheat. The 3'-untranslated region of *ScCbf14 *was not sequenced in this study; it would be interesting to sequence this region to investigate whether it also contains SNPs significantly associated with FT in rye as well. However, members of the *Cbf *gene family are not the only key factors in the frost responsive network [55,56]. Hannah et al. [10] reported that 45% of the *Arabidopsis *transcriptome was cold responsive, but only 33% of the cold responsive transcriptome belonged to the *Cbf *regulon and in a study of wheat, Monroy et al. [13] reported that at least one-third of genes induced by cold did not belong to the *Cbf *regulon. The transcription factor *AtHOS9*, which encodes a putative homeobox protein, has been shown to contribute to the regulation of FT in *Arabidopsis *independently of the *Cbf *regulon [57]. Thus, extending our research to more candidate genes of the frost responsive network will certainly be worthwhile.

The *Dreb2 *gene, another member of the *AP2 *transcription factor family, has been isolated and characterized in several crop species such as wheat, barley, maize, and rice [58-61]. Similar to *Cbf *genes, *Dreb2 *can specifically bind to DRE/CRT *cis*-elements of the stress-inducible target genes, albeit primarily under drought rather than cold/frost stress [62]. However, it is not surprising that *Dreb2 *can also be induced by cold/frost as shown by recent studies in wheat and maize since both drought and cold/frost stresses lead to dehydration of cells [59,60]. In this study, three SNPs in *ScDreb2 *were significantly associated with FT supporting the hypothesis that *Dreb2 *in rye is not only involved in drought response but also in frost response.

The dehydrin genes, part of the *COR *gene family, are regulated by the *Cbf *gene family and the *Dreb2 *gene via the *cis*-element DRE/CRT present in the promoter region of *COR *genes [19]. Transcripts of *HvDhn1*, *HvDhn3 *and other *HvDhn *genes were detected under frost stress in barley [18]. We detected two SNPs in the promoter region of *ScDhn1 *and one SNP in the intron of *ScDhn3 *with significant associations with FT in the controlled platform. These SNPs might serve as variants which affect the binding specificity of the *Cbf *gene family.

Effect sizes of markers, commonly expressed as percentage of the genetic variance explained by markers, are of primary interest in association studies since they are the main factors that determine the effectiveness of subsequent marker assisted-selection processes. Two hypotheses for the distribution of effect sizes in quantitative traits have been proposed: Mather' s "infinitesimal" model and Robertson's model [63]. The former assumes an effectively infinitesimal number of loci with very small and nearly equal effect sizes; the latter, an exponential trend of the distribution of effects whereby a few loci have relatively large effects and the rest only small effects. Findings in this study support the latter, with distributions of SNP effect sizes (percentage of the genetic variance explained by individual SNPs) highly concentrated near zero and few SNPs having large effects (maximum 28.8% explained genetic variation). A similar distribution of haplotype effect sizes was observed. A recent review summarizing association studies in 15 different plant species also implicated Robertson's model and further suggested that phenotypic traits, species, and types of variants may impact distributions of effect sizes [64]. Studies on the genetic architecture of quantitative traits have become a challenging task in recent years [64-66]. We will further investigate this topic with a genome-wide association study to obtain a more complete picture of the genetic architecture of FT.

Epistasis, generally defined as the interaction between genes, has been recognized for over a century [67], and recently it has been suggested that it should be explicitly modeled in association studies in order to detect "missing heritabilities" [68,69]. Several recent association studies in plants have revealed the presence of epistasis in complex traits, including potato tuber quality, barley flowering time, and maize kernel quality [70-72]. In this study, eleven, six, and one significant (*P *< 0.05) gene × gene interaction effects were found in the controlled, semi-controlled and field platforms, respectively, suggesting that epistasis may play a role in the frost responsive network. From the frost responsive network, one might hypothesize that transcription factors interact with their downstream target genes, for example, that *ScIce2 *interacts with the *ScCbf *gene family and the latter interacts with *COR *genes, such as the dehydrin (*Dhn*) gene family. Indeed significant interactions were observed between *ScIce2 *× *ScCbf15*, *ScCbf14 *× *ScDhn3*, and *ScDreb2 *× *ScDhn3*. Some candidate genes in the same cascade level also interact with each other, such as members of the *ScCbf *gene family *ScCbf6 *× *ScCbf15 *and *ScCbf11 *× *ScCbf14*. Similar interactions within the *Cbf *gene family were also observed in *Arabidopsis *where *AtCbf2 *was indicated as a negative regulator of *AtCbf1*and *AtCbf3*[73]. In this study, *ScVrn1 *was not significantly associated with FT but had significant interaction effects with six other candidate genes, underlining the important role of *ScVrn1 *in the frost responsive network. To confirm direct physical interactions of transcription factors with their downstream target genes, further experiments are needed, for example, electrophoresis mobility shift assays or ChIP (chromatin immunoprecipitation) sequencing technology. It is worth to point out that the power of detecting gene × gene interaction might be low due to relative small sample size.

Low to moderate empirical correlations of SNP-FT associations were observed across the three platforms reflecting the complexity of FT and thus the need for different platforms in order to more accurately characterize FT. There are at least two reasons that might explain why relatively low to medium empirical correlations of SNP-FT associations were observed: 1) different duration and intensity of freezing temperature and 2) different levels of confounding effects from environmental factors other than frost stress *per se*. In the controlled platform, plants were cold-hardened and then exposed to freezing temperatures (-19°C or -21°C) in a short period of six days using defined temperature profiles. Recovery score in the controlled platform represents the most pure and controlled measurement of FT among the three platforms since the effect of environmental factors other than frost stress is minimized. In the semi-controlled platform, plants were exposed to much longer freezing periods with fluctuating temperatures and repeated frost-thaw processes. In addition, a more complex situation occurs in this platform, requiring plants to cope with other variable climatic factors such as changing photoperiod, natural light intensity, wind, and limited water supply. Thus, the measurement % plants with undamaged leaves in the semi-controlled platform reflects the combined effect of various environmental influences and stresses on the vitality of leaf tissue but does not mirror survival of the crown tissue as indicator for frost tolerance. In the field platform, winter temperatures were generally lower than in the semi-controlled platform due to the strong continental climate in Eastern Europe and Canada (Additional file 1). The measurement % survival in the field is further confounded by environmental effects, such as snow-coverage, soil uniformity, topography, and other unmeasured factors. The different experimental platforms permit the identification of different sets of genes associated with FT, which might impact the correlations of SNP-FT associations across platforms. It is worth to point out that the correlation between the controlled and semi-controlled platform was higher than between the semi-controlled and field platform. One possible explanation is that plant growth in boxes in both controlled and semi-controlled platforms results in a rather similar environment where roots are more exposed to freezing than in the field. Several studies suggested that different genes might be induced under different frost stress treatments. A large number of blueberry genes induced in growth chambers were not induced under field conditions [74]. In rye, Campoli et al. [24] drew the conclusion that expression patterns of different members of the *Cbf *gene family were affected by different acclimation temperatures and sampling times. Most prior studies on FT have been conducted in controlled environments. However, the relatively low to medium correlation among platforms in this study suggest that future studies should consider various scenarios in order to obtain a more complete picture of the genetic basis of FT in rye.

## Conclusions

Identification of alleles and genes underlying agronomic traits such as FT is important for genome-based breeding. Based on phenotypic data from three different phenotyping platforms, including field trials, our study showed that the *Cbf *gene family plays an important role in FT of rye. Nine out of twelve candidate genes that had previously been shown to be directly involved in the cold/frost responsive network were significantly associated with FT. Several significant gene × gene interactions were observed indicating the presence of epistatic interactions between genes involved in the frost responsive network. Our results demonstrated that the candidate gene-based association approach remains one of the most appropriate strategies for gene identification, given the huge genome size of rye (~8,100 Mb) and the rapid decline of linkage disequilibrium (LD) revealed in a previous study [43]. Validation of SNPs and haplotypes associated with FT will be performed in future studies to determine the diagnostic value of markers for marker-assisted selection in rye breeding programs.

## Authors' contributions

YL carried out the candidate gene and population structure analysis, participated in the statistical analyses and drafted the manuscript. AB carried out statistical analyses. GH participated in the molecular analyses and interpretation of the results. DA participated in statistical analysis and interpretation of the results. VK provided SSR marker data. PW developed the plant material. EB and CCS designed and coordinated the study and interpreted the results. All authors read, edited and approved the final manuscript.

## Supplementary Material

Additional file 1 **Geographical coordinates and climate data for semi-controlled and field platforms**. The file contains geographical coordinates of the experimental stations, dates of sowing and scoring, and temperature during the trial period in the semi-controlled and field platforms. Click here for file

Additional file 2 **Supplementary information on primers and sequence analysis for *ScDreb2***. The file contains two tables. Table S1 describes the primer information of *ScDreb2*. Table S2 is a summary of the *ScDreb2 *sequence analysis including analyzed fragment length, gene coverage, number of lines, number of SNPs (MAF > 0.05), number of Indels and haplotypes, haplotype (*Hd*) and nucleotide diversity (*π*), and linkage disequilibrium (LD). Click here for file

Additional file 3 **Full information of SNP-FT associations**. The file contains allelic effect (*β_SNP_*), SNP effect (% genetic variation explained), and *P*-value of 170 SNPs associated with FT in controlled, semi-controlled, and field platforms. Click here for file
