# Supplementary material for: Association analysis of frost tolerance in rye using candidate genes and phenotypic data from controlled, semi-controlled, and field phenotyping platforms
Source: BMC Plant Biol. 2011 Oct 27;11:146. doi: 10.1186/1471-2229-11-146 (PMC3228716; doi:10.1186/1471-2229-11-146)
Supplement: Additional file 1 — Geographical coordinates and climate data for semi-controlled and field platforms. The file contains geographical coordinates of the experimental stations, dates of sowing and scoring, and temperature during the trial period in the semi-controlled and field platforms. [file 1471-2229-11-146-S1.PDF]

Additional file 1. Geographical coordinates, sowing and scoring dates, and climate data for semi-controlled and field platforms

Locations	Geographical coordinates	Dates of sowing [YYYY-MM-DD]	Dates of scoring [YYYY-MM-DD]	Temperature (°C)[1]		
				Average	Min	Max
Oberer Lindenhof, Germany	48°46'N, 09°10'E	2007-09-01	2008-01-16 / 2008-02-20 / 2008-04-01	3.5	-17.0	23.0
		2008-09-23	2009-02-05 / 2009-03-16	2.5	-19.4	25.1
Kasan, Russia	55°38'N, 49°18'E	2008-09-10[2]	2009-04-27	-3.5	-31	20
Lipezk, Russia	52°47'N, 39°01'E	2008-09-18[2]<br>2009-09-08[2]	2009-04-08 /<br>2010-04-06	-1.8<br>-3.6	-23<br>-31	16<br>23
Minsk, Belarus	54°06'N, 28°17'E	2008-09-22	2009-04-NA	NA	-20.4	25.7
Saskatoon1, Canada<br>Saskatoon2, Canada	52°08'N,106°40'W	2008-08-29<br>2008-09-02	2009-04-30<br>2009-04-30	-12.6	-41.4	20.6

[1] Location and year:	Climate data recording period:	Data source:
Oberer Lindenhof 2007/2008:	2007-09-01 to 2008-03-31	Experimental station Oberer Lindenhof
Oberer Lindenhof 2008/2009:	2008-09-01 to 2009-03-31	Experimental station Oberer Lindenhof
Kasan 2008/2009:	2008-10-01 to 2009-04-27	Weather station at Bol'shiye Kabany near experimental station
Lipezk 2008/2009:	2008-10-17 to 2009-04-08	http://www.tutiempo.net/en/Climate/ELEC/08-2009/279280.htm<br>weather station is ca. 40 km away from the experimental station
Lipezk 2009/2010:	2009-10-01 to 2010-04-06	see above
Minsk 2008/2009:	2008-09-01 to 2009-04-31	http://www.tutiempo.net/en/Climate/BORISOV/06-2011/267590.htm<br>weather station is ca. 20 km away from the experimental station
Saskatoon 1 and 2, 2008/2009:	2008-12-01 to 2009-04-30	http://www.climat.meteo.gc.ca , data for Saskatoon RCS (52°10'N, 106°43'W)

[2] Scoring of the number of plants before winter was done 3-4 weeks after sowing. Climate data are reported for the time between scoring before and after winter.

NA: Data not available
