# Supplementary material for: Association analysis of frost tolerance in rye using candidate genes and phenotypic data from controlled, semi-controlled, and field phenotyping platforms
Source: BMC Plant Biol. 2011 Oct 27;11:146. doi: 10.1186/1471-2229-11-146 (PMC3228716; doi:10.1186/1471-2229-11-146)
Supplement: Additional file 2 — Supplementary information on primers and sequence analysis for ScDreb2. The file contains two tables. Table S1 describes the primer information of ScDreb2. Table S2 is a summary of the ScDreb2 sequence analysis including analyzed fragment length, gene coverage, number of lines, number of SNPs (MAF > 0.05), number of Indels and haplotypes, haplotype (Hd) and nucleotide diversity (π), and linkage disequilibrium (LD). [file 1471-2229-11-146-S2.PDF]

# Additional file 2

**Table S1 Primer information for *ScDreb2***

| Primer set name | Forward and reverse primer sequence (5'-3') | Analysed sequence length (bp) | Gene coverage | Sequence for primer design (GenBank accession number) | Annealing temperature (°C) | Taq DNA polymerase supplier | GenBank accession number |
|---|---|---|---|---|---|---|---|
| *ScDreb2*- fragment 1 | F: TGGAGCAGAGGAAAGTACCCGGA<br>R: AGGTGGCTTCCTCGCCCTCT | 919 | Exon 3 and intron 3 | GU017675 (Aegilops) | 65 | QIAGEN | HQ730774 |
| *ScDreb2*- fragment 2 | F: CCAGCCTGGAAGGTGAGATCTTCTGT<br>R: ATAGATGCCACTGGCGGCGCA | 758 | Exon 4 | GU017675 (Aegilops) | 58 | QIAGEN | HQ730774 |

PCR was performed according to our previous study:

Li Y, Haseneyer G, Schön CC, Ankerst D, Korzun V, Wilde P, Bauer E: **High levels of nucleotide diversity and fast decline of linkage disequilibrium in rye (*Secale cereale* L.) genes involved in frost response.** *BMC Plant Biology* 2011, 11:6.

**Table S2 Summary information of candidate gene (CG) *ScDreb2*: Analyzed fragment length, gene coverage, number of lines, number of SNPs (MAF>0.05), number of Indels and haplotypes, haplotype (*Hd*) and nucleotide diversity (*π*), and linkage disequilibrium (LD)**

| CG | Fragment length (bp) | Gene coverage [a] | No. of lines [b] | No. of SNPs (non-synonymous) | No. of Indels | No. of haplotypes | *Hd* ± SD | $\pi \pm SD \times 10^{-3}$ (only exon) | Intra-genic LD ($r^2$) |
|---|---|---|---|---|---|---|---|---|---|
| *ScDreb2* | 1677 | E3/I3/E4 | 197 | 13 (4) | 0 | 88 | 0.89 ± 0.02 | 3.0 ± 0.3 (3.4 ± 0.1) | 0.06 |

[a] E: exon; I: intron
[b] Failure of amplification in 4 lines may be due to the presence of SNPs/Indels in the primer binding sites of these sequence
