# Supplementary material for: Association analysis of frost tolerance in rye using candidate genes and phenotypic data from controlled, semi-controlled, and field phenotyping platforms
Source: BMC Plant Biol. 2011 Oct 27;11:146. doi: 10.1186/1471-2229-11-146 (PMC3228716; doi:10.1186/1471-2229-11-146)
Supplement: Additional file 3 — Full information of SNP-FT associations. The file contains allelic effect (βSNP), SNP effect (% genetic variation explained), and P-value of 170 SNPs associated with FT in controlled, semi-controlled, and field platforms. [file 1471-2229-11-146-S3.PDF]

Additional file 3 Allelic effect (β_{SNP}), SNP effect (% genetic variation explained), and *P*-value of 170 SNPs associated with frost tolerance in three phenotyping platforms

	Controlled (recovery score 0-5)			Semi-controlled (% plants with undamaged leaves)			Field (% survival)		
Gene_SNP	β_{SNP}	% variation	*P*- value	β_{SNP}	% variation	*P*- value	β_{SNP}	% variation	*P*- value
*ScCbf2*_SNP1	-0.26	27.88	**6.27E-05**[a]	-0.18	1.04	0.7803	0.75	0.00	0.5021
*ScCbf2*_SNP2	0.02	0.13	0.7591	0.18	2.23	0.7046	-0.20	0.00	0.7925
*ScCbf2*_SNP3	0.17	0.00	**0.0236**	2.17	5.32	**0.0022**	1.59	0.74	0.1904
*ScCbf6*_SNP1	0.03	1.15	0.6609	0.88	11.11	0.1040	1.33	3.89	0.1492
*ScCbf6*_SNP2	-0.04	0.00	0.4681	0.51	7.03	0.3075	0.76	2.79	0.3714
*ScCbf6*_SNP3	-0.05	0.00	0.3833	0.26	4.43	0.6162	0.78	2.60	0.3685
*ScCbf9b*_SNP1	0.05	1.85	0.3110	-0.32	3.06	0.4807	0.29	0.39	0.7038
*ScCbf9b*_SNP2	0.05	1.67	0.3516	-0.23	2.30	0.6116	-0.05	0.09	0.9449
*ScCbf9b*_SNP3	0.04	1.30	0.3945	-0.22	2.11	0.6294	-0.22	0.00	0.7756
*ScCbf9b*_SNP4	0.04	0.00	0.4512	-0.14	2.15	0.7584	-0.22	0.07	0.7712
*ScCbf9b*_SNP5	-0.04	0.00	0.5591	0.79	5.76	0.1994	-0.13	0.03	0.8995
*ScCbf9b*_SNP6	-0.12	0.00	0.0995	-0.28	0.67	0.6726	-1.27	4.61	0.2407
*ScCbf9b*_SNP7	-0.17	0.00	0.0726	0.59	1.90	0.4474	-1.17	1.88	0.3569
*ScCbf9b*_SNP8	-0.43	22.81	**1.74E-05**	-1.37	8.85	0.1303	-1.16	7.98	0.4407
*ScCbf9b*_SNP9	-0.25	22.69	**0.0005**	-0.84	10.68	0.2000	-0.33	0.56	0.7688

Gene_SNP	Controlled (recovery score 0-5)			Semi-controlled (% plants with undamaged leaves)			Field (% survival)		
	β_{SNP}	% variation	*P*- value	β_{SNP}	% variation	*P*- value	β_{SNP}	% variation	*P*- value
*ScCbf9b*_SNP10	0.04	0.02	0.5407	0.34	0.00	0.5319	0.80	0.83	0.3883
*ScCbf9b*_SNP11	-0.28	23.77	**0.0003**	-0.99	11.70	0.1748	-0.80	4.29	0.5159
*ScCbf9b*_SNP12	-0.23	22.35	**0.0013**	-0.80	10.52	0.2253	-0.72	1.42	0.5240
*ScCbf9b*_SNP13	-0.24	19.09	**0.0013**	-0.87	10.69	0.2027	-0.59	1.42	0.6132
*ScCbf9b*_SNP14	0.05	0.00	0.3195	0.59	0.00	0.2086	-0.42	0.00	0.6048
*ScCbf9b*_SNP15	0.12	0.00	**0.0485**	0.39	0.00	0.4522	-0.36	0.00	0.6768
*ScCbf9b*_SNP16	-0.28	26.24	**0.0002**	-1.27	14.65	0.0746	-1.14	5.05	0.3469
*ScCbf9b*_SNP17	-0.43	23.52	**6.33E-06**	-0.94	9.18	0.2879	0.07	3.65	0.9652
*ScCbf9b*_SNP18	-0.01	0.21	0.9008	-0.07	0.39	0.8828	-0.59	0.15	0.4528
*ScCbf9b*_SNP19	-0.10	0.00	0.2814	0.54	1.50	0.4763	-1.92	10.18	0.1353
*ScCbf9b*_SNP20	0.04	0.48	0.5532	0.65	0.00	0.2327	0.51	0.00	0.5727
*ScCbf9b*_SNP21	0.03	0.92	0.6007	0.23	0.87	0.6857	-0.54	2.75	0.5794
*ScCbf9b*_SNP22	0.03	0.41	0.6751	0.22	0.45	0.6966	-0.88	5.32	0.3510
*ScCbf9b*_SNP23	-0.28	26.24	**0.0002**	-1.27	14.65	0.0746	-1.14	5.05	0.3469
*ScCbf9b*_SNP24	-0.03	0.00	0.7040	0.72	4.03	0.2732	-1.00	3.59	0.3433
*ScCbf9b*_SNP25	-0.26	18.52	**0.0003**	-0.84	9.46	0.1695	-1.11	4.51	0.2713
*ScCbf9b*_SNP26	-0.24	8.26	**0.0004**	-0.17	0.53	0.7819	-1.94	19.93	0.0653

Gene_SNP	Controlled (recovery score 0-5)			Semi-controlled (% plants with undamaged leaves)			Field (% survival)		
	β_{SNP}	% variation	*P*- value	β_{SNP}	% variation	*P*- value	β_{SNP}	% variation	*P*- value
*ScCbf9b*_SNP27	-0.18	16.37	**0.0002**	0.67	0.00	0.1487	-0.94	6.14	0.2160
*ScCbf9b*_SNP28	-0.16	0.00	0.0995	0.81	5.62	0.3534	0.53	1.68	0.7004
*ScCbf9b*_SNP29	0.02	0.41	0.7932	0.43	1.29	0.5030	-0.98	4.00	0.3708
*ScCbf9b*_SNP30	-0.05	0.00	0.5568	0.84	0.00	0.2205	-1.16	3.90	0.3102
*ScCbf9b*_SNP31	-0.22	13.79	**0.0002**	-0.14	0.81	0.7864	2.02	3.01	**0.0222**
*ScCbf11*_SNP1	0.18	0.00	0.0523	1.82	0.00	**0.0456**	-0.64	2.01	0.6609
*ScCbf11*_SNP2	0.18	0.00	0.0523	1.82	0.00	**0.0456**	-0.64	2.01	0.6609
*ScCbf11*_SNP3	0.18	0.00	0.0523	1.82	0.00	**0.0456**	-0.64	2.01	0.6609
*ScCbf11*_SNP4	0.18	0.00	0.0523	1.82	0.00	**0.0456**	-0.64	2.01	0.6609
*ScCbf11*_SNP5	0.18	0.00	0.0523	1.82	0.00	**0.0456**	-0.64	2.01	0.6609
*ScCbf11*_SNP6	0.18	0.00	0.0523	1.82	0.00	**0.0456**	-0.64	2.01	0.6609
*ScCbf11*_SNP7	0.18	0.00	0.0523	1.82	0.00	**0.0456**	-0.64	2.01	0.6609
*ScCbf11*_SNP8	0.06	0.00	0.5495	1.57	0.00	0.0760	-1.04	3.93	0.4689
*ScCbf11*_SNP9	0.10	0.00	0.2802	1.30	0.00	0.1314	-1.09	4.73	0.4359
*ScCbf11*_SNP10	0.06	0.00	0.5495	1.57	0.00	0.0760	-1.04	3.93	0.4689
*ScCbf11*_SNP11	0.06	0.00	0.5495	1.57	0.00	0.0760	-1.04	3.93	0.4689
*ScCbf11*_SNP12	0.10	0.00	0.2802	1.30	0.00	0.1314	-1.09	4.73	0.4359

Gene_SNP	Controlled (recovery score 0-5) β_{SNP}	% variation	*P*- value	Semi-controlled (% plants with undamaged leaves) β_{SNP}	% variation	*P*- value	Field (% survival) β_{SNP}	% variation	*P*- value
*ScCbf11*_SNP13	-0.08	4.06	0.1882	-0.52	7.64	0.3597	1.11	5.85	0.2496
*ScCbf11*_SNP14	-0.06	3.46	0.3113	-0.29	5.80	0.6062	1.13	7.64	0.2403
*ScCbf11*_SNP15	0.06	0.00	0.5495	1.57	0.00	0.0760	-1.04	3.93	0.4689
*ScCbf11*_SNP16	0.06	0.00	0.5495	1.57	0.00	0.0760	-1.04	3.93	0.4689
*ScCbf11*_SNP17	-0.06	3.46	0.3113	-0.29	5.80	0.6062	1.13	7.64	0.2403
*ScCbf11*_SNP18	-0.06	3.46	0.3113	-0.29	5.80	0.6062	1.13	7.64	0.2403
*ScCbf11*_SNP19	-0.06	3.46	0.3113	-0.29	5.80	0.6062	1.13	7.64	0.2403
*ScCbf11*_SNP20	-0.06	3.46	0.3113	-0.29	5.80	0.6062	1.13	7.64	0.2403
*ScCbf11*_SNP21	-0.06	3.46	0.3113	-0.29	5.80	0.6062	1.13	7.64	0.2403
*ScCbf11*_SNP22	-0.06	3.46	0.3113	-0.29	5.80	0.6062	1.13	7.64	0.2403
*ScCbf11*_SNP23	-0.06	3.46	0.3113	-0.29	5.80	0.6062	1.13	7.64	0.2403
*ScCbf11*_SNP24	-0.06	3.46	0.3113	-0.29	5.80	0.6062	1.13	7.64	0.2403
*ScCbf11*_SNP25	-0.06	3.46	0.3113	-0.29	5.80	0.6062	1.13	7.64	0.2403
*ScCbf11*_SNP26	-0.06	3.46	0.3113	-0.29	5.80	0.6062	1.13	7.64	0.2403
*ScCbf11*_SNP27	-0.04	1.68	0.5515	0.06	0.40	0.9196	1.58	8.13	0.1013
*ScCbf12*_SNP1	-0.32	0.00	**2.01E-05**	-1.79	0.00	**0.0142**	-0.64	0.00	0.6029
*ScCbf12*_SNP2	-0.33	1.33	**3.49E-06**	-2.01	0.00	**0.0037**	-0.28	0.00	0.8069

Gene_SNP	Controlled (recovery score 0-5)			Semi-controlled (% plants with undamaged leaves)			Field (% survival)		
	β_{SNP}	% variation	*P*- value	β_{SNP}	% variation	*P*- value	β_{SNP}	% variation	*P*- value
*ScCbf12*_SNP3	-0.36	0.00	**1.01E-05**	-1.36	1.40	0.0861	0.23	2.81	0.8637
*ScCbf12*_SNP4	-0.30	4.36	**1.01E-05**	-1.69	0.00	**0.0138**	-0.60	0.00	0.5964
*ScCbf12*_SNP5	0.17	12.53	**0.0059**	-0.11	1.02	0.8503	-0.36	5.91	0.7158
*ScCbf12*_SNP6	-0.31	0.00	**0.0030**	-1.44	0.00	0.1278	3.89	28.84	**0.0140**
*ScCbf12*_SNP7	-0.16	1.43	0.0824	-0.92	6.36	0.3004	-1.11	0.00	0.4501
*ScCbf12*_SNP8	-0.25	3.59	**0.0008**	-1.40	2.98	0.0525	0.39	1.47	0.7373
*ScCbf12*_SNP9	-0.32	15.91	**1.07E-06**	-1.18	0.00	0.0665	-0.46	0.72	0.6616
*ScCbf12*_SNP10	-0.14	0.00	0.0882	-0.58	2.48	0.4686	-0.36	0.00	0.7861
*ScCbf12*_SNP11	-0.20	0.00	**0.0137**	-1.14	3.02	0.1498	-1.69	0.00	0.1971
*ScCbf12*_SNP12	-0.23	2.10	**0.0046**	-1.30	4.07	0.0989	-1.84	0.00	0.1584
*ScCbf12*_SNP13	-0.21	1.88	**0.0110**	-1.45	2.84	0.0723	-2.12	0.00	0.1093
*ScCbf12*_SNP14	-0.19	0.64	**0.0323**	-1.29	7.40	0.1366	-1.33	0.00	0.3438
*ScCbf12*_SNP15	-0.23	6.92	**0.0017**	-0.65	0.66	0.3550	-0.56	0.00	0.6387
*ScCbf12*_SNP16	-0.36	11.93	**6.02E-07**	-1.97	0.00	**0.0046**	-0.80	0.00	0.4852
*ScCbf12*_SNP17	-0.26	5.91	**0.0108**	-2.15	14.34	**0.0292**	-3.66	6.71	**0.0232**
*ScCbf12*_SNP18	-0.23	13.46	**0.0023**	-1.65	7.18	**0.0274**	-1.18	0.67	0.3507
*ScCbf12*_SNP19	-0.28	8.15	**5.31E-05**	-1.67	1.59	**0.0137**	-0.23	0.00	0.8362

Gene_SNP	Controlled (recovery score 0-5) β_{SNP}	% variation	*P*- value	Semi-controlled (% plants with undamaged leaves) β_{SNP}	% variation	*P*- value	Field (% survival) β_{SNP}	% variation	*P*- value
*ScCbf12*_SNP20	-0.17	4.38	**0.0084**	0.15	0.00	0.8056	0.02	0.16	0.9826
*ScCbf12*_SNP21	-0.18	0.86	**0.0321**	-0.27	0.00	0.7461	-0.43	0.00	0.7550
*ScCbf12*_SNP22	-0.19	0.00	**0.0245**	-1.17	5.24	0.1678	-0.93	0.00	0.5026
*ScCbf12*_SNP23	0.07	0.00	0.4320	-1.41	2.70	0.0891	1.01	2.21	0.4563
*ScCbf12*_SNP24	-0.01	0.24	0.9506	-2.44	17.42	**0.0056**	-0.59	0.00	0.6869
*ScCbf12*_SNP25	-0.13	1.09	0.0714	-0.47	0.00	0.4932	-0.94	0.21	0.3696
*ScCbf12*_SNP26	0.11	2.29	0.0671	-0.10	0.15	0.8559	-1.99	11.81	**0.0369**
*ScCbf14*_SNP1	-0.06	0.28	0.5108	-0.56	1.65	0.4813	-1.65	9.51	0.2086
*ScCbf14*_SNP2	-0.06	0.28	0.5108	-0.56	1.65	0.4813	-1.65	9.51	0.2086
*ScCbf14*_SNP3	-0.09	2.11	0.2836	-0.51	1.67	0.5414	-1.78	10.53	0.1937
*ScCbf14*_SNP4	-0.06	0.28	0.5108	-0.56	1.65	0.4813	-1.65	9.51	0.2086
*ScCbf14*_SNP5	-0.08	1.16	0.3520	-0.37	1.53	0.6633	-1.49	9.26	0.2846
*ScCbf15*_SNP1	-0.13	5.07	**0.0391**	-1.34	4.77	**0.0192**	-3.15	7.26	**0.0008**
*ScCbf15*_SNP2	-0.14	0.00	**0.0166**	-1.34	1.42	**0.0112**	-3.20	9.60	**0.0002**
*ScCbf15*_SNP3	-0.22	5.71	**0.0009**	-0.74	3.64	0.2066	-2.32	3.91	**0.0181**
*ScCbf15*_SNP4	-0.06	0.00	0.3770	-0.93	5.99	0.1319	-1.27	0.00	0.2226
*ScDhn1*_SNP1	-0.14	0.24	0.1269	0.31	0.00	0.6911	-3.18	0.79	**0.0292**

Gene_SNP	Controlled (recovery score 0-5)			Semi-controlled (% plants with undamaged leaves)			Field (% survival)		
	β_{SNP}	% variation	*P*- value	β_{SNP}	% variation	*P*- value	β_{SNP}	% variation	*P*- value
*ScDhn1*_SNP2	-0.22	0.00	**0.0143**	-0.24	0.00	0.7710	-3.60	1.66	**0.0198**
*ScDhn1*_SNP3	-0.22	0.00	**0.0200**	0.68	3.95	0.4302	-3.36	0.00	**0.0399**
*ScDhn1*_SNP4	-0.11	0.00	0.2968	0.49	25.61	0.5610	-2.49	0.00	0.1401
*ScDhn1*_SNP5	-0.06	0.00	0.4529	0.18	4.21	0.8051	-1.74	0.90	0.1830
*ScDhn1*_SNP6	-0.15	0.00	0.0556	0.43	10.19	0.5497	-1.94	1.70	0.1363
*ScDhn3*_SNP1	-0.03	1.02	0.7531	-1.06	0.00	0.2100	-1.02	7.03	0.4763
*ScDhn3*_SNP2	0.10	0.13	0.4100	-1.31	6.59	0.2101	0.82	0.00	0.6225
*ScDhn3*_SNP3	0.25	1.64	**0.0237**	-1.50	0.00	0.1699	0.87	0.00	0.6020
*ScDhn3*_SNP4	0.14	2.41	0.2748	-1.83	0.00	0.1397	1.37	0.00	0.4661
*ScDhn3*_SNP5	-0.04	0.00	0.4219	0.07	1.02	0.8888	-0.70	0.00	0.4302
*ScDhn3*_SNP6	0.00	0.00	0.9543	0.21	1.77	0.6854	0.14	0.28	0.8737
*ScDhn3*_SNP7	-0.03	0.00	0.5362	0.12	1.01	0.8160	-0.01	0.00	0.9871
*ScDhn3*_SNP8	-0.02	0.00	0.6552	0.28	0.81	0.5980	0.01	0.11	0.9880
*ScDhn3*_SNP9	0.01	0.89	0.8497	0.00	0.14	0.9970	0.52	2.20	0.5616
*ScDhn3*_SNP10	0.01	0.00	0.8827	-0.89	1.81	0.3017	-1.38	4.67	0.3199
*ScDhn3*_SNP11	0.08	0.00	0.3551	-0.63	1.86	0.4641	-0.83	2.23	0.5544
*ScDhn3*_SNP12	-0.33	3.22	0.0699	-1.20	4.34	0.3861	-0.91	0.00	0.7074

	Controlled (recovery score 0-5)			Semi-controlled (% plants with undamaged leaves)			Field (% survival)		
Gene_SNP	β_{SNP}	% variation	*P*- value	β_{SNP}	% variation	*P*- value	β_{SNP}	% variation	*P*- value
*ScDhn3*_SNP13	-0.22	3.99	0.2188	-1.95	1.11	0.1810	-0.11	0.03	0.9645
*ScDhn3*_SNP14	0.09	0.00	0.3031	0.30	0.00	0.7135	0.44	0.00	0.7463
*ScDreb2*_SNP1	-0.02	0.88	0.6928	0.31	0.00	0.5587	1.02	1.65	0.2375
*ScDreb2*_SNP2	-0.08	3.53	0.3600	-1.13	0.00	0.1688	-1.17	0.46	0.4328
*ScDreb2*_SNP3	-0.16	1.26	**0.0179**	-0.69	0.00	0.2620	-2.02	2.38	0.0611
*ScDreb2*_SNP4	-0.05	0.00	0.4379	-0.25	0.96	0.6359	-1.07	1.27	0.2328
*ScDreb2*_SNP5	-0.12	0.00	0.0629	-0.36	0.00	0.5449	-1.91	2.37	0.0580
*ScDreb2*_SNP6	-0.12	0.00	0.0741	-0.27	0.00	0.6538	-1.44	2.23	0.1648
*ScDreb2*_SNP7	-0.05	0.00	0.3442	-0.19	2.07	0.6998	-1.25	3.55	0.1343
*ScDreb2*_SNP8	-0.01	0.00	0.9245	-0.72	0.71	0.4195	-0.30	0.75	0.8463
*ScDreb2*_SNP9	0.00	0.05	0.9575	0.18	0.80	0.7372	0.44	0.00	0.6507
*ScDreb2*_SNP10	0.16	0.00	0.0801	0.15	0.00	0.8639	-0.09	0.74	0.9530
*ScDreb2*_SNP11	-0.28	14.59	**0.0004**	-0.21	0.00	0.7771	-0.44	0.57	0.7330
*ScDreb2*_SNP12	-0.13	3.13	0.1026	0.69	3.57	0.3525	-2.60	5.17	**0.0327**
*ScDreb2*_SNP13	-0.05	1.66	0.6374	-0.28	0.24	0.7880	-1.13	1.80	0.5105
*ScIce2*_SNP1	0.29	13.67	**0.0003**	0.41	0.00	0.5777	3.75	15.46	**0.0022**
*ScIce2*_SNP2	0.32	14.71	**6.69E-05**	0.18	0.00	0.8014	3.37	10.01	**0.0055**

	Controlled (recovery score 0-5)			Semi-controlled (% plants with undamaged leaves)			Field (% survival)		
Gene_SNP	β_{SNP}	% variation	*P*- value	β_{SNP}	% variation	*P*- value	β_{SNP}	% variation	*P*- value
*ScIce2*_SNP3	0.32	14.71	**6.69E-05**	0.18	0.00	0.8014	3.37	10.01	**0.0055**
*ScIce2*_SNP4	0.25	12.76	**0.0019**	0.14	0.00	0.8513	3.37	10.01	**0.0055**
*ScIce2*_SNP5	0.30	6.05	**0.0008**	1.43	0.00	0.0722	3.44	7.30	**0.0093**
*ScIce2*_SNP6	0.28	5.81	**0.0017**	1.56	0.00	0.0512	4.13	12.20	**0.0018**
*ScIce2*_SNP7	0.32	5.85	**0.0059**	1.22	0.00	0.1285	4.30	14.30	**0.0014**
*ScIce2*_SNP8	0.30	6.05	**0.0008**	1.43	0.00	0.0722	3.44	7.30	**0.0093**
*ScIce2*_SNP9	0.30	6.05	**0.0008**	1.43	0.00	0.0722	3.44	7.30	**0.0093**
*ScIce2*_SNP10	0.30	6.05	**0.0008**	1.43	0.00	0.0722	3.44	7.30	**0.0093**
*ScIce2*_SNP11	0.30	6.05	**0.0008**	1.43	0.00	0.0722	3.44	7.30	**0.0093**
*ScIce2*_SNP12	0.30	6.05	**0.0008**	1.43	0.00	0.0722	3.44	7.30	**0.0093**
*ScIce2*_SNP13	0.25	12.46	**0.0024**	0.27	0.00	0.7152	3.19	10.42	**0.0095**
*ScIce2*_SNP14	0.30	6.05	**0.0008**	1.43	0.00	0.0722	3.44	7.30	**0.0093**
*ScIce2*_SNP15	0.25	12.46	**0.0024**	0.27	0.00	0.7152	3.19	10.42	**0.0095**
*ScIce2*_SNP16	0.25	12.46	**0.0024**	0.27	0.00	0.7152	3.19	10.42	**0.0095**
*ScIce2*_SNP17	0.25	12.46	**0.0024**	0.27	0.00	0.7152	3.19	10.42	**0.0095**
*ScIce2*_SNP18	0.30	6.05	**0.0008**	1.43	0.00	0.0722	3.44	7.30	**0.0093**
*ScIce2*_SNP19	-0.14	0.00	**0.0074**	0.44	3.21	0.3770	-0.82	0.00	0.3229

Gene_SNP	Controlled (recovery score 0-5)			Semi-controlled (% plants with undamaged leaves)			Field (% survival)		
	β_{SNP}	% variation	*P*- value	β_{SNP}	% variation	*P*- value	β_{SNP}	% variation	*P*- value
*ScIce2*_SNP20	-0.14	0.65	0.1187	0.64	1.18	0.4897	-0.60	0.00	0.7030
*ScIce2*_SNP21	-0.11	0.00	**0.0322**	0.44	1.50	0.3771	-1.03	0.00	0.2070
*ScIce2*_SNP22	-0.14	0.00	**0.0039**	0.37	3.31	0.4433	-1.01	0.00	0.2091
*ScIce2*_SNP23	-0.07	0.00	0.1674	0.54	4.49	0.2659	-0.90	0.00	0.2627
*ScIce2*_SNP24	0.10	0.00	0.0548	0.01	0.23	0.9849	0.18	0.00	0.8297
*ScIce2*_SNP25	-0.11	0.00	**0.0433**	0.15	0.58	0.7633	-0.55	0.00	0.5058
*ScIce2*_SNP26	-0.13	0.00	**0.0292**	-0.02	0.01	0.9686	-0.62	0.00	0.4936
*ScIce2*_SNP27	-0.12	0.00	**0.0177**	0.21	2.40	0.6755	-1.19	0.00	0.1508
*ScIce2*_SNP28	-0.10	0.00	0.0600	0.30	3.31	0.5643	-1.00	0.00	0.2366
*ScIce2*_SNP29	-0.11	0.00	**0.0425**	0.12	1.12	0.8222	-0.86	0.00	0.3115
*ScIce2*_SNP30	-0.07	0.00	0.1582	0.54	3.62	0.2710	-0.71	0.00	0.3846
*ScIce2*_SNP31	-0.11	0.00	**0.0205**	0.52	4.91	0.2824	-0.86	0.00	0.2792
*ScIce2*_SNP32	-0.05	0.00	0.3082	1.12	5.08	**0.0167**	-0.52	0.00	0.5053
*ScIce2*_SNP33	-0.02	0.05	0.7024	0.80	11.03	0.0711	-0.99	0.00	0.1708
*ScIce2*_SNP34	0.00	0.00	0.9524	1.02	12.75	**0.0218**	-0.84	0.00	0.2475
*ScIce2*_SNP35	-0.01	0.01	0.8347	0.44	4.39	0.3415	-0.93	0.00	0.2166
*ScIce2*_SNP36	-0.03	0.00	0.4900	0.95	8.54	**0.0328**	-0.76	0.00	0.2996

	Controlled (recovery score 0-5)			Semi-controlled (% plants with undamaged leaves)			Field (% survival)		
Gene_SNP	β_{SNP}	% variation	*P*- value	β_{SNP}	% variation	*P*- value	β_{SNP}	% variation	*P*- value
*ScIce2*_SNP37	0.03	0.00	0.4396	1.28	10.00	**0.0035**	-0.60	0.00	0.4055
*ScVrn1*_SNP1	0.04	0.00	0.6560	-1.01	0.06	0.2520	-0.64	0.16	0.6600

[a] *P*-values < 0.05 are printed in bold
